# Knowledge of healthcare providers in the management of anaphylaxis

**DOI:** 10.1016/j.waojou.2021.100599

**Published:** 2021-11-09

**Authors:** Sandra Nora González-Díaz, Rosalaura Virginia Villarreal-González, Elma I. Fuentes-Lara, María del Rocío Salinas-Díaz, Cindy Elizabeth de Lira-Quezada, Carlos Macouzet-Sánchez, Alejandra Macías-Weinmann, Rosa Ivett Guzmán-Avilán, Mariano García-Campa

**Affiliations:** Autonomous University of Nuevo Leon. University Hospital “Dr. José Eleuterio González”, Regional Center of Allergy and Clinical Immunology, Monterrey, Nuevo León, Mexico

**Keywords:** Anaphylaxis, Epinephrine, Healthcare providers, Knowledge

## Abstract

**Introduction:**

Anaphylaxis is defined as a severe, life-threatening systemic hypersensitivity reaction. Early diagnosis and treatment of a severe allergic reaction requires recognition of the signs and symptoms, as well as classification of severity. It is a clinical emergency, and healthcare providers should have the knowledge for recognition and management. The aim of the study is to evaluate the level of knowledge in the management of anaphylaxis in healthcare providers.

**Methods:**

It is an observational, descriptive, cross-sectional study conducted among healthcare providers over 18 years old via a Google Forms link and shared through different social media platforms. A 12-item questionnaire was applied which included the evaluation of the management of anaphylaxis, from June 2020 to May 2021.

**Results:**

A total of 1023 surveys were evaluated; 1013 met inclusion criteria and were included in the statistical analysis. A passing grade was considered with 8 or more correct answers out of 12; the overall approval percentage was 28.7%. The group with the highest percentage of approval in the questionnaire was health-care providers with more than 30 years of work experience. There was a significant difference between the proportions of approval between all specialty groups, and in a post-hoc analysis, allergy and immunology specialists showed greater proportions of approval compared to general medicine practitioners (62.9% vs 25%; *p=<0.001*).

**Conclusions:**

It is important that healthcare providers know how to recognize, diagnose, and treat anaphylaxis, and later refer them to specialists in Allergy and Clinical Immunology in order to make a personalized diagnosis and treatment.

## Introduction

Anaphylaxis is defined as a severe, life-threatening systemic hypersensitivity reaction characterized by sudden onset, with respiratory and cardiovascular symptoms, and usually presenting with skin symptoms.^1^^,^^2^

Prevalence of anaphylaxis varies widely and many studies suggest that it is increasing, particularly in developed countries; it is estimated between 1.6% and 5.1%.^3^, ^4^, ^5^ Available data indicate that anaphylaxis is underrecognized and undertreated in the United States. Furthermore, evidence indicates that the majority of patients at high risk for anaphylactic events are not receiving prescriptions for epinephrine autoinjectors in a timely manner.^6^ Studies have shown that a large percentage of patients (57%) who present to the emergency room (ER) with anaphylaxis can be misdiagnosed. Moreover, even when correctly diagnosed, epinephrine, the essential first line in the treatment of anaphylaxis, is frequently (up to 80% of the time) not administered.^7^ In addition to being underdiagnosed, anaphylaxis is undertreated in the ERs, and many patients who should be carrying epinephrine autoinjectors are not receiving prescriptions for them.^6^

Anaphylaxis occurs as a combination of symptoms that can affect several organs and systems, from mild to moderate and even progressing to death, and accounts for up to 0.26% of overall hospital admissions.^8^ Despite an increase in hospitalizations over time due to anaphylaxis, mortality remains low, estimated at 0.05–0.51 per million people/year for drugs, 0.03–0.32 for food, and 0.09–0.13 for hymenoptera venom-induced anaphylaxis.^9^^,^^10^ It is a clinical emergency that healthcare providers should know how to rapidly manage and treat.^11^

Anaphylaxis is a serious systemic hypersensitivity reaction that is usually rapid in onset and may cause death. Severe anaphylaxis is characterized by potentially life-threatening compromise in airway, breathing, and/or circulation, and may occur without typical skin features or circulatory shock being present. Furthermore, the World Allergy Organization (WAO) Anaphylaxis Committee has proposed to amend the current NIAID/FAAN criteria: 1. Typical skin symptoms AND significant symptoms from at least 1 other organ system; OR 2. Exposure to a known or probable allergen for that patient, with respiratory and/or cardiovascular compromise.^1^ The use of tryptase levels is an objective and useful parameter in certain situations such as distinguishing the differential diagnosis with other conditions such as septic shock, anaphylaxis in surgery setting, and when the signs and symptoms can overlap with drug effects. On the other hand, identifying base tryptase level would help to recognize patients at risk of future anaphylaxis recurrence, such as clonal mast cell disorders or hereditary alpha tryptasemia syndrome.^1^

The first-line treatment for anaphylaxis is intramuscular epinephrine and should be administered immediately.^1^^,^^12^ Risk factors that increase the severity of anaphylaxis include the delay in the use of epinephrine, followed by the severity of the reaction, absence of urticaria, biphasic reaction, use of beta-blockers or angiotensin converting enzyme inhibitors, age over 65 years, cardiovascular or lung disease, uncontrolled asthma, drug-induced anaphylaxis, elevated serum tryptase, and platelet activating factor deficiency.^12^^,^^13^

Solé et al^14^ reported in a study about patients who were seen by allergists from July 2008 to June 2010 in Latin American countries and Portugal that most patients (80.5%) had acute severe allergic epsiodes treated in an emergency setting while the rest remained at the place where the reaction occurred. Isolated or associated medications that were used to treat acute episodes were recognized by 63.9% of the patients and included systemic corticosteroids (oral or injectable) in 80.5% of the patients, antihistamines (oral or injectable) in 70.2%, and epinephrine (subcutaneous or intramuscular) in 37.3%. Jares et al^15^ described 273 patients with drug-induced anaphylaxis and only 27% received epinephrine. Prompt epinephrine administration is essential treatment of anaphylaxis, and delay in management can lead to fatal consequences. ^1^^,^^12^

Early diagnosis and treatment of severe allergic reaction requires recognition of the signs and symptoms. The aim of the study is to evaluate the level of knowledge in the management of anaphylaxis in healthcare providers.

## Methodology

An observational, descriptive, cross-sectional study was conducted among healthcare providers over 18 years old via a Google Forms link and shared through different social media platforms. Digital informed consent was provided by all survey participants prior to their enrollment. Participants who accepted digital informed consent and answered all the questions were included in the study. The surveys were eliminated in case participants did not accept digital informed consent. The study was submitted and approved by the Bioethics and Safety Committee with registration code AL20-00008. A total of 1023 surveys were collected from June 2020 to May 2021.

A 12 item-questionnaire was applied (Table 1), which included the evaluation of the management of anaphylaxis. Demographic variables such as gender, age, place of residence, academic degree, years of work experience, and area of work were evaluated. To safeguard the security and confidentiality of the participants, the data were coded.

**Table 1:** Questionnaire of knowledge in the management of anaphylaxis

Q1	Gell & Coombs classification for hypersensitivity reaction in Anaphylaxis
Q2	First-line treatment in anaphylaxis
Q3	Second-line treatment in anaphylaxis
Q4	Third-line treatment in anaphylaxis
Q5	Dose and concentration of epinephrine during anaphylaxis
Q6	Maximum therapeutic dose of epinephrine in adults during anaphylaxis
Q7	Maximum therapeutic dose of epinephrine in children during anaphylaxis
Q8	Preferred route of administration during an episode of anaphylaxis
Q9	Time interval between the application of subsequent doses of epinephrine
Q10	Anatomical position during anaphylaxis episode
Q11	Time of surveillance recommended after mild-moderate anaphylaxis
Q12	Time of surveillance recommended after severe anaphylaxis

Data analysis was performed using SPSSv20IBM Corp. (Released 2011. IBM SPSS Statistics for Windows, Version 20.0). Frequencies and percentages were reported for qualitative variables and measures of central tendency and dispersion for quantitative variables. The distribution of the sample was evaluated by the Kolmogorov-Smirnov test. Pearson's Chi-square test was used to evaluate categorical variables where, in tests of more than 2 groups, a post-hoc study was carried out based on the standardized residuals with the Bonferroni correction to the value of p 15; the values of *p = 0.05* was considered significant for the rest of the tests.

## Results

**Fig. 1:**
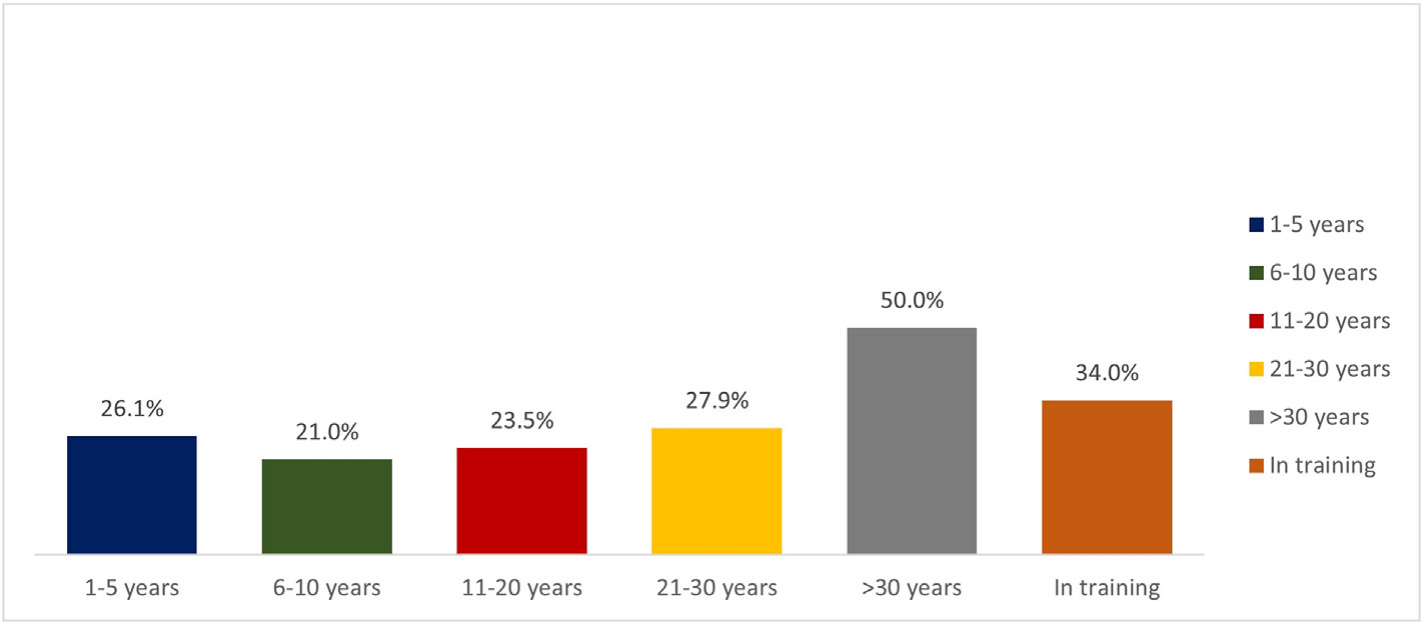
Percentage of approved questionnaire by years of work experience.

A total of 1023 surveys were evaluated; 10 participants did not accept the digital informed consent. There were 1013 who met inclusion criteria and were included in the statistical analysis. The majority of participants were women 68.3% (n = 692). They were analyzed by groups of years of work experience: 1–5 years with 33.3%, 6–10 years with 15.5%, 11–20 years with 13.4%, 21–30 years with 4.3%, more than 30 years of work experience with 5.9%, and healthcare providers such as fellows in training including medical, dental, and nursing students, corresponded to 27.6% (Table 2). The group with the highest percentage of correct answers in the questionnaire were healthcare providers with more than 30 years of work experience (Fig. 1).

**Table 2:** Distribution of response to the questionnaire by years of work experience.

Question Correct	1–5 years n = 337 (%)	6–10 years n = 157 (%)	11–20 years n = 136 (%)	21–30 years n = 43 (%)	>30 years n = 60 (%)	In training n = 279 (%)	P Value	
Q1	239 (70.9%)	95 (60.5%)^a^	83 (61%)^a^	32 (74.4%)	49 (81.7%)	223 (80.0%)	<0.001	1^a^
Q2	250 (74.2%)	105 (66.9%)^a^	86 (63.2%)^a^	32 (74.4%)	55 (91.7%)	233 (83.5%)	<0.001	0.540^a^
Q3	176 (52.2%)	76 (48.4%)	67 (49.2%)	21 (48.8%)	32 (53.3%)	171 (61.3%)	0.075	
Q4	88 (26.1%)	44 (28.0%)	32 (23.5%)	12 (27.9%)	24 (40.0%)	71 (25.4%)	0.255	
Q5	139 (41.2%)	76 (48.4%)	66 (48.5%)	16 (37.2%)	35 (58.3%)	128 (45.9%)	0.122	
Q6	138 (40.9%)	65 (41.4%)	64 (47.1%)	26 (60.5%)^a^	37 (61.7%)^a^	138 (49.4%)	0.008	1^a^
Q7	134 (38.9%)	60 (38.2%)	60 (44.1%)	18 (41.9%)	38 (63.3%)^a^	145 (52%)^a^	0.001	0.118^a^
Q8	228 (67.7%)	92 (58.6%)	72 (52.9%)^a^	27 (18.9%)	45 (75.0%)	240 (86.0%)^a^	<0.001	<0.001^a^
Q9	217 (64.4%)	96 (61.1%)	88 (64.7%)	17 (39.5%)^a^	30 (50.0%)^a^	181 (64.9%)	0.010	0.322^a^
Q10	113 (33.5%)^a^	66 (42.0%)	53 (39.0%)	19 (44.2%)	36 (60.0%)^a^	101 (36.2%)	0.004	<0.001^a^
Q11	140 (41.5%)	54 (34.4%)	59 (43.4%)	22 (51.2%)	26 (43.3%)	120 (43.0%)	0.363	
Q12	88 (31.2%)	33 (19.7%)^a^	32 (28.7%)	12 (34.9%)	30 (26.7%)	95 (34.1%)^a^	0.048	0.001^a^

a Post-hoc analysis based on standarized residuals. After Bonferroni Correction, for statistical difference p-value was considered as p = 0.004

**Fig. 2:**
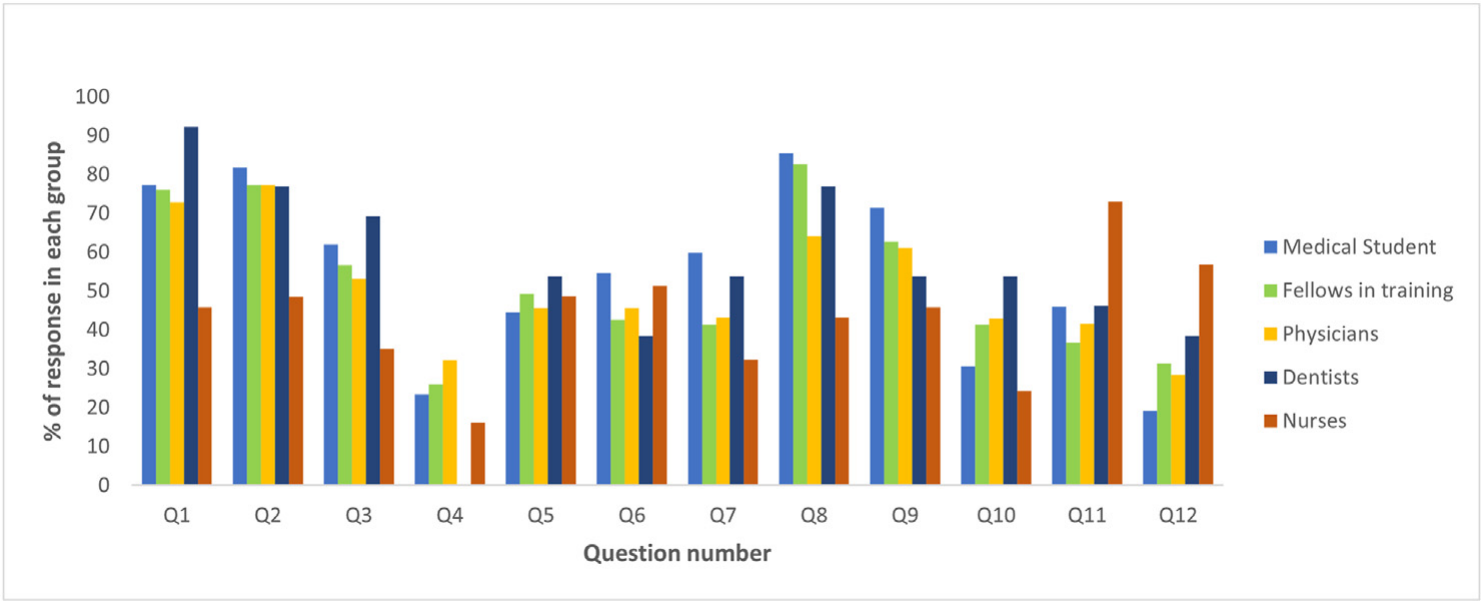
Percentage of response to the questionnaire by healthcare providers.

**Fig. 3:**
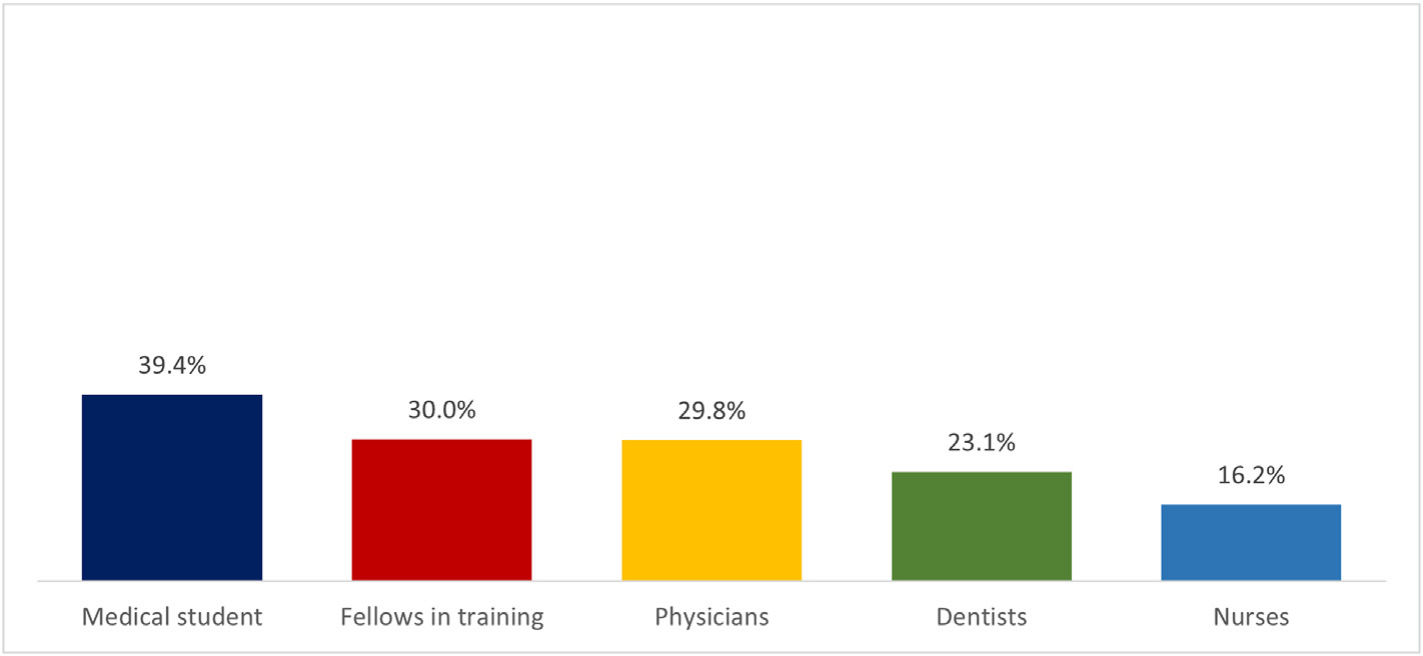
Percentage approved of the questionnaire by healthcare providers.

Of the 1013 surveys of healthcare providers, 79.3% correspond to those who are in charge of management during an episode of anaphylaxis, such as physicians, fellows in training, medical students, dentists, and nurses. The remaining 20.7% corresponds to other allied health professions. (Table 3, Fig. 2). Medical students were the group with the highest percentage of correct answers in the questionnaire (Fig. 3).

**Table 3:** Distribution of response to the questionnaire by healthcare providers.

Questions		Medical Student (n = 137)	Fellows in training (n = 150)	Physicians (n = 466)	Dentists (n = 13)	Nurses (n = 37)	Total (n = 803)	*P*-value	
	Q1								
Correct		106 (77.3%)	114 (76.0%)	340 (72.9%)	12 (92.3%)^a^	17 (45.9%)^a^	589	<0.001	0.003^a^
Not correct		31 (22.7%)	36 (24.0%)	126 (27.1%)	1 (7.7%)^a^	20 (54.1%)^a^	214		
	Q2								
Correct		112 (81.8%)^a^	116 (77.3%)	360 (77.3%)	10 (76.9%)	18 (48.6%)^a^	616	<0.001	<0.001^a^
Not correct		25 (18.2%)^a^	34 (22.7%)	106 (22.7%)	3 (23.1%)	19 (51.4%)^a^	187		
	Q3								
Correct		85 (62.1%)	85 (56.7%)	248 (53.2%)	9 (69.2%)	13 (35.1%)	440	0.032	0.004^a^
Not correct		52 (37.9%)	65 (43.3%)	218 (46.8%)	4 (30.7%)	24 (64.9%)	363		
	Q4								
Correct		32 (23.4%)	39 (26.0%)	150 (32.2%)^a^	0 (0.0%)^a^	6 (16.2%)	227	0.010	0.012^a^
Not correct		105 (76.6%)	111 (74.0%)	316 (67.8%)^a^	13 (100.0%)^a^	31 (83.8%)	576		
	Q5								
Correct		61 (44.5%)	74 (49.3%)	213 (45.7%)	7 (53.8%)	18 (48.7%)	373	0.884	
Not correct		76 (55.5%)	76 (50.7%)	253 (54.3%)	6 (46.2%)	19 (51.3%)	430		
	Q6								
Correct		75 (54.7%)	64 (42.7%)	213 (45.7%)	5 (38.5%)	19 (51.3%)	376	0.249	
Not correct		62 (45.3%)	86 (57.3%)	253 (54.3%)	8 (61.5%)	18 (48.7%)	427		
	Q7								
Correct		82 (59.9%)^a^	62 (41.3%)	202 (43.3%)	7 (53.8%)	12 (32.4%)^a^	365	0.003	0.004^a^
Not correct		22 (16.1%)^a^	88 (58.7%)	264 (56.7%)	6 (46.2%)	25 (67.6%)^a^	438		
	Q8								
Correct		117 (85.4%)^a^	124 (82.7%)	299 (64.2%)	10 (76.9%)	16 (43.2%)^a^	566	0.000	<0.001^a^
Not correct		20 (14.6%)^a^	26 (17.3%)	167 (35.8%)	3 (23.1%)	21 (56.8%)^a^	237		
	Q9								
Correct		98 (71.5%)^a^	94 (62.7%)	285 (61.2%)	7 (53.8%)	17 (45.9%)^a^	501	0.043	0.005^a^
Not correct		39 (28.5%)^a^	56 (37.3%)	181 (38.8%)	6 (46.2%)	20 (54.1%)^a^	302		
	Q10								
Correct		42 (30.7%)^a^	62 (41.3%)	200 (42.9%)	7 (53.8%)	9 (24.3%)^a^	320	0.021	0.544^a^
Not correct		95 (69.3%)^a^	88 (58.7%)	266 (57.1%)	6 (46.2%)	28 (75.7%)^a^	483		
	Q11								
Correct		63 (46.0%)	55 (36.7%)^a^	194 (41.6%)	6 (46.2%)	27 (73.0%)^a^	345	0.002	<0.001^a^
Not correct		74 (54.0%)	95 (63.3%)^a^	272 (58.4%)	7 (53.8%)	10 (27.0%)^a^	458		
	Q12								
Correct		49 (19.2%)	47 (31.3%)	133 (28.5%)	5 (38.5%)	21 (56.8%)	255	0.059	
Not correct		88 (16.1%)	103 (68.7%)	333 (71.5%)	8 (61.5%)	16 (43.2%)	548		
Aproved		54 (39.4%)	45 (30.0%)	139 (29.8%)	3 (23.1%)	6 (16.2%)	247	0.059	

a Post-hoc analysis based on standarized residuals. After Bonferroni Correction, for statistical difference p-value was considered as p = 0.005

Most of the participants work in outpatient consult 64.8%, followed by hospitalization 27.7%, the emergency room 11.7%, and intensive care unit 1.1%.

The average approved percentage of the survey by profession was 32.3%: 25% in general practitioners, 23.1% dentists, 23.5% nurses, and the specialty with the highest approved percentage was Allergy and Immunology with 62.9%. (Table 4). A total of 399 healthcare providers with a medical specialty participated: 140 from Allergy and Immunology, 123 from Pediatrics, 57 from Anesthesiology, 29 from Oncology, 21 from Internal Medicine, 11 from Emergencies, 10 from General Surgery, 6 from Hematology, and 2 from Intensive Therapy, resulting in a higher percentage approved of the survey by Allergy and Immunology specialists with 62.9%, followed by Intensive therapy 50%, and Pediatrics 32.2%.

**Table 4:** Percentage approved of the questionnaire by healthcare profession.

Medical speciality	Approval (%)	p-value	
Medical Doctor	75 (25.0)^a^	<0.001	<0.001^a^
Pediatrics	39 (32.2)		
Internal Medicine	3 (14.3)		
Emergency	0 (0.0)		
Anesthesiology	13 (22.8)		
Surgery	3 (30.0)		
Intensive Therapy	1 (50.0)		
Allergy & Immunology	88 (62.9)^a^		
Hematology	1 (16.7)		
Oncology	4 (14.8)		
Dentists	3 (23.1)		
Nurses	4 (23.5)		
Total approved	234 (32.3)		

a Post-hoc analysis based on standardized residuals. After Bonferroni Correction, for statistical difference p-value was considered as p = 0.002

**Fig. 4:**
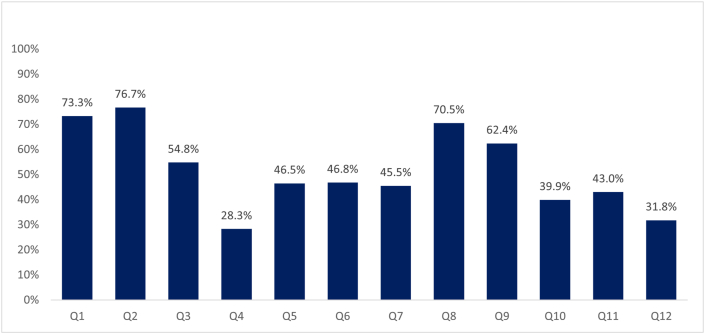
Percentage of correct answers for question in healthcare providers.

Of the 1013 surveys, 71.3% answered that anaphylaxis is a type 1 hypersensitivity reaction, 75.2% answered that the first-line treatment in anaphylaxis is epinephrine, 53% responded that the second-line treatment for anaphylaxis is fluid therapy, and 26.7% responded that the third-line treatment is steroids and antihistamines. There were 45.5% of the participants who responded that the epinephrine dose is 0.01 mg/kg/dose in 1:1000 concentration; 56.0% who responded that the preferred route of administration of epinephrine is intramuscular, 62.1% who responded that the interval time between the application of a subsequent doses of adrenaline is 5–10 min, and 38.3% who responded that Trendelenburg is the anatomical position in which to place a patient with anaphylaxis. A passing grade was considered with 8 or more correct answers out of 12; the overall approval percentage was 28.7% (Fig. 4).

Based on the academic grade of the population, a significant difference was found in the proportions of questions 1–4 and 7–11. After the Bonferroni correction, the value of p = 0.005 was taken into account as significant for the post-hoc analysis where in question 1 the proportions between the group of dentists and the nursing staff were statistically significant (92.3% vs 45.9%; *p = 0.003*).

In question 2, the significant difference was marked by the groups of medical students and nurses, where the former had a higher proportion of correct answers (81.8% vs 48.6*%; p= <0.001*). Similarly, medical students yielded higher proportions of correct answers in question 3 (62.1% vs 35.1%; *p=0.004*) as well as in question 7 (59.9% vs 32.4%; *p=0.004*) and in question 8 (85.4% vs 43.2%; *p=<0.001*). In question 11, the nursing personnel yielded higher proportions of correct responses compared to specialty residents (73% vs 36.7%; *p=<0.001*). No significant differences were found between the group's exam approval.

When divided by years of experience in their clinical field, significant differences between the groups was found in questions 1–2, 6–9, 11 and the proportion of the exam approval. In the post-hoc analyses, in training (fellow in training, medical, dentists and nurses students) had higher proportions compared to physicians with 11–20 years of experience in question 8 (86% vs 52.9%; *p=<0.001*). In question 10, physicians with more than 30 years of experience demonstrated higher proportions of correct answers compared to physicians with 1–5 years of experience (60% vs 33.5%; *p= <0.001*). Finally, medical students presented higher proportions in correct answers in question 12 compared to physicians with 6–10 years of experience (34.1% vs 19.7%; *p=0.001*). There was a significant difference between the proportions of approval between all specialty groups and in a post-hoc analysis, allergy and immunology specialists showed greater proportions of approval compared to general medicine practitioners (62.9% vs 25%; *p=<0.001*).

## Discussion

Anaphylaxis is a serious allergic reaction and a condition that requires immediate initial treatment; delay in management can lead to fatal consequences.^1^^,^^10^

Current guidelines for the management of anaphylaxis recommend the use of epinephrine as first-line treatment for anaphylaxis.^18^ In previous studies where knowledge about anaphylaxis was evaluated, it was reported that most of the participants used epinephrine as their first-line treatment. In 2013, Grossman et al^16^ conducted a survey of 1114 participants and divided the years of work experience of healthcare personnel: <5 years (8.9%), 5–10 years (21.9%), 11–20 years (44.8%), and more than 20 years (24.4%); in comparison with our study 1–5 years (33.3%), 6–10 years (15.5%), 11–20 years 13.4%, 21–30 years (4.3%), and more than 30 years of work experience 5.9%, and physician in training corresponded to 27.6%.

In our study, the group with more than 30 years of experience as well as the medical students had a higher percentage of correct answers. These differences could be due to an increase in the number of publications on anaphylaxis in recent years, better clinical training, or the inclusion of the topic in conferences, academic sessions, and in the academic syllabus.

The staff responded that the treatment for anaphylaxis is epinephrine with 93.6% and the preferred intramuscular route in 66.9%.^15^

Grossman et al^16^ found that 93.6% of the participants used epinephrine as their first-line treatment,^15^ similar to reported by Prabhu et al^17^ in 90% of the participants; in our study, 75% of health-care providers answered that epinephrine is the first-line treatment for anaphylaxis. Most of the physicians in our study know that adrenaline is the first-line treatment for anaphylaxis, however some physicians do not. We have to continue creating awareness in adrenaline use and the importance of not delaying it. Epinephrine has a rapid onset of action and acts at different levels: increasing cardiac output, blood pressure and reducing mucosal edema, reducing airway resistance and slowing the progression of the allergic reaction. Although there are second- and third-line therapies such as corticosteroids and antihistamines, these treatments do not provide the benefits that adrenaline offers.

Drupad et al^18^ in 2015, conducted a study on 265 healthcare providers among medical students and nursing staff. Within the results, 65.7% were correct in that anaphylaxis is a type 1 hypersensitivity reaction compared to our study with 71.3%, epinephrine as the first line of treatment in 65.7% vs. 75.2% in our study, the correct dose of epinephrine in 20.4% compared to our study with 45.5%, and the intramuscular route of administration in 31.7% vs. 56.0% in our study, having a higher percentage of correct answers in our healthcare staff.

Olabarri et al^19^ conducted a study on 425 pediatric emergency care providers with a mean age of 28 years, and reported that 99.7% of their healthcare providers would apply epinephrine as the first line of treatment for anaphylaxis, 92.6% by an intramuscular route, 86.1% anterolateral thigh application site, and 81.6% the correct dose of epinephrine. They report that these physicians would refer these patients to a specialist in Allergy and Immunology in 69.4% of cases, compared to what was reported in our study, which was 92.3%.

Pimental et al^20^ reported 196 specialist physicians: pediatrics, internal medicine, cardiology, anesthesiology, general surgery, orthopedics, and gynecology. In 72.4% of the physicians, they responded that adrenaline is the first line of treatment, intramuscular route in 64.3%, correct dose in 50%; while other studies report that 70.9% of healthcare providers do not know the correct dose in an acute episode of anaphylaxis.^21^

Hypersensitivity reactions such as anaphylaxis are a public health problem, so strategies need to be planned to promote training for healthcare providers in the management of these reactions. It is important that healthcare providers such as primary care physicians, medical students, nurses, dentists, emergency physicians, intensive care medical doctors, and anesthesiologists diagnose and treat anaphylaxis, and later refer them to specialists in Allergy and Clinical Immunology in order to make a personalized diagnosis and treatment. The numerous barriers regarding the proper and timely identification of anaphylaxis remain a problem. The results observed in our study reinforce the need to increase all the necessary measures to improve the diffusion of clinical criteria in order to enhance the identification of the condition and to proceed with the treatment, especially in primary care physicians.

## Abbreviations

CORP, Corporation; ER, Emergency room; IBM, International Business Machines; Q, Question; SPSS, Statistical Package for the Social Sciences; WAO, World Allergy Organization.

## Funding

The authors declare that no funding was received for the present study.

## Ethical statement

This study was approved by the clinical research ethics committee of the University Hospital and Faculty of Medicine of the Autonomous University of Nuevo Leon in Mexico before the initiation of this study. Participants have signed written informed consent.

## Consent

All authors consent for publication.

## Authors' contributions

SNGD, RVVG, design of the study, manuscript elaboration and revision. CELQ, EIFL and MRSD contributed to design of the study, data collection, interpretation of the results and manuscript writing. CMS, AMW and RIGA contributed to data collection. EIFL and RVVG manuscript writing an data collection as well. MGC performed analysis and interpretation of results. All authors aided in interpreting the results and worked on the manuscript. All authors discussed the results and commented on the manuscript. All authors read and approved the final manuscript. We confirm that the manuscript has been read and approved by all named authors.

## Data availability

Additional data is available upon request.

## Declaration of competing interest

The authors declare they have no conflicts of interest to disclose.
